# Improvement of glycine biosynthesis from one‐carbon compounds and ammonia catalyzed by the glycine cleavage system in vitro

**DOI:** 10.1002/elsc.202100047

**Published:** 2021-11-14

**Authors:** Yingying Xu, Jie Ren, Wei Wang, An‐Ping Zeng

**Affiliations:** ^1^ Beijing Advanced Innovation Center for Soft Matter Science and Engineering Beijing University of Chemical Technology Beijing P. R. China; ^2^ State Key Laboratory for Biology of Plant Diseases and Insect Pests/Key Laboratory of Control of Biological Hazard Factors (Plant Origin) for Agri‐product Quality and Safety Ministry of Agriculture Institute of Plant Protection Chinese Academy of Agricultural Sciences Beijing P. R. China; ^3^ Institute of Bioprocess and Biosystems Engineering, Hamburg University of Technology Hamburg Germany

**Keywords:** C1 assimilation, glycine cleavage system, glycine synthesis, H protein, in vitro metabolic engineering

## Abstract

Glycine cleavage system (GCS) plays a central role in one‐carbon (C1) metabolism and receives increasing interest as a core part of the recently proposed reductive glycine pathway (rGlyP) for assimilation of CO_2_ and formate. Despite decades of research, GCS has not yet been well understood and kinetic data are barely available. This is to a large degree because of the complexity of GCS, which is composed of four proteins (H, T, P, and L) and catalyzes reactions involving different substrates and cofactors. In vitro kinetics of reconstructed microbial multi‐enzyme glycine cleavage/synthase system is desired to better implement rGlyP in microorganisms like *Escherichia coli* for the use of C1 resources. Here, we examined in vitro several factors that may affect the rate of glycine synthesis via the reverse GCS reaction. We found that the ratio of GCS component proteins has a direct influence on the rate of glycine synthesis, namely higher ratios of P protein and especially H protein to T and L proteins are favorable, and the carboxylation reaction catalyzed by P protein is a key step determining the glycine synthesis rate, whereas increasing the ratio of L protein to other GCS proteins does not have significant effect and the ratio of T protein to other GCS proteins should be kept low. The effect of substrate concentrations on glycine synthesis is quite complex, showing interdependence with the ratios of GCS component proteins. Furthermore, adding the reducing agent dithiothreitol to the reaction mixture not only results in great tolerance to high concentration of formaldehyde, but also increases the rate of glycine synthesis, probably due to its functions in activating P protein and taking up the role of L protein in the non‐enzymatic reduction of H_ox_ to H_red_. Moreover, the presence of some monovalent and divalent metal ions can have either positive or negative effect on the rate of glycine synthesis, depending on their type and their concentration.

**Abbreviations**
5,10‐CH_2_‐THF: N^5^,N^10^‐methylene‐THF
DNSCl: dansyl chloride
DTT: dithiothreitol
*E. coli*
: *Escherichia coli*
GCS: glycine cleavage system or glycine synthase
H_int_
: aminomethylated form of the lipoylated H protein
H_ox_
: oxidized form of the lipoylated H protein
H_red_
: reduced form of the lipoylated H protein
PLP: pyridoxal 5‐phosphate monohydrate
rGlyP: reductive glycine pathway
THF: tetrahydrofolate

## 1 INTRODUCTION

Nowadays, most of the chemicals including fuels, solvents and plastics are produced from fossil carbons. However, with the imminent depletion of fossil carbons and the concomitant increase in atmospheric CO_2_, the use of other more abundant carbon sources to replace fossil carbons as the prime source of fuels and value‐added chemicals has become an urgent issue. It has been reported that bioprocesses account for merely 3.5% of the current production of commodity and specialty chemicals [1], partly because the use of simple sugars and starches directly competes with human consumption and hence can undermine food security and decrease biodiversity. There is an urgent need to search for sustainable and cheap resources as feedstocks for the production of high‐value chemicals at hundreds of millions of tons per year using microbial fermentation processes.

**FIGURE 1:**
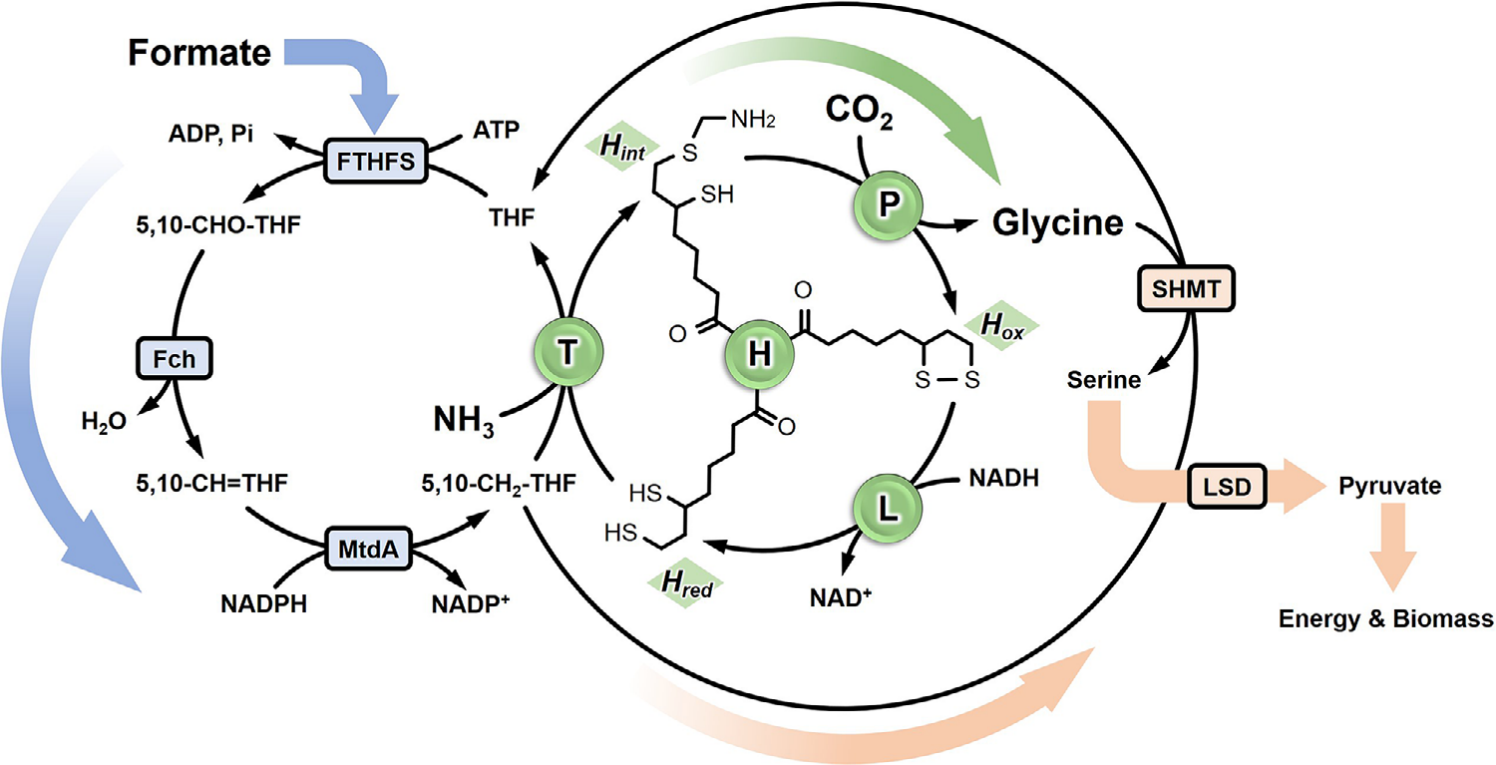
Schematic diagram of the reductive glycine pathway (rGlyP) in close relation to the THF cycle. Enzyme abbreviations: FTHFS refers to formate tetrahydrofolate ligase (EC 6.3.4.3″, Fch to methenyltetrahydrofolate cyclohydrolase (EC 3.5.4.9), MtdA to methylenetetrahydrofolate dehydrogenase (EC 1.5.1.5), SHMT to serine hydroxymethyltransferase (EC 2.1.2.1), LSD to L‐serine dehydratase (EC 4.3.1.17), P to pyridoxal 5′‐phosphate‐dependent glycine dehydrogenase (EC 1.4.4.2), T to tetrahydrofolate‐dependent aminomethyltransferase (EC 2.1.2.10), H to lipoamide‐containing aminomethylene carrier, L to NAD^+^‐dependent lipoamide dehydrogenase (EC 1.8.1.4). The colored arrows indicate the three modules of the rGlyP, namely the assimilation of formate to form 5,10‐CH_2_‐THF (blue arrow), the synthesis of glycine by the reverse GCS reaction (green arrow), and the channeling of glycine into the central carbon metabolism (pink arrow)

One‐carbon (C1) compounds, such as CO_2_ [2, 3], formate [4, 5] and methanol [6, 7] have been proposed as ideal feedstocks to alleviate global energy shortage and environmental pollution problems. These compounds are either naturally abundant, cheap to produce, or available as industrial by‐products. In particular, C1 compounds produced from CO_2_ and renewable energy (e.g., formate generated from CO_2_ via electrochemical synthesis [8, 9]) are attractive alternative carbon sources. Only a small group of anaerobic microbes can assimilate C1 compounds natively. However, the high cost of cultivation and technique limitation constrains the microbial industry production from C1 compounds. Therefore, reprogramming C1 metabolic pathway in industrial model microorganisms such as *E. coli* and yeast has become a choice to deal with such problems. Bar‐Even et al. [10] proposed the reductive glycine pathway (rGlyP) (Figure 1) as an appealing pathway in the use of formate and CO_2_ for biosynthesis. The rGlyP has several advantages, such as oxygen tolerance of the enzymes involved, high energy efficiency, and independent operation with little overlapping with the central metabolism. In the rGlyP, formate is ligated to tetrahydrofolate (THF) and further reduced to N^5^, N^10^‐methylene‐THF (5,10‐CH_2_‐THF), which is used for the synthesis of glycine by glycine synthase (the reverse glycine cleavage system, rGCS). Glycine is then condensed with another molecular 5,10‐CH_2_‐THF catalyzed by serine hydroxymethyltransferase (SHMT) to produce serine. Finally, the deamination of serine catalyzed by serine deaminase yields pyruvate, which enters the central metabolic pathway.

Recently, this novel C1 assimilation pathway has been discovered to naturally exist in the sulphate‐reducing bacterium *Desulfovibrio desulfuricans* [11], the phosphate‐oxidizing bacterium *Candidatus phosphitivorax* [12], and the acetogen *Clostridium drakei* [13]. It has also been successfully constructed in industrial strains like the model bacterium *E. coli* [14, 15] for the use of formate and CO_2_. Theoretically, the rGlyP can replace the glycolytic pathway in microorganisms by only use formate or CO_2_ as substrate. However, the efficiency of such synthetic rGlyP is still so low that it cannot yet meet the needs of practical applications. Very recently, through short‐term evolution, Kim et al. [16] improved the growth of an *E. coli* strain containing the rGlyP on formate and CO_2_ alone, resulting in a reduction of the doubling time from about 70 h to less than 8 h and a growth yield of ∼2.3 g of cell dry weight (gCDW) per mol of formate. Bang et al. [17] engineered *E. coli* to grow on CO_2_ and formate by introducing the rGlyP, so that the engineered strain can grow to an optical density at 600 nm of 7.38 in 450 h. Efforts have also been made to implement the rGlyP into other microorganisms. Hong et al. [18] has introduced GCS coding genes from *Gottschalkia acidurici* into *Clostridium pasteurianum* and demonstrated thereby assimilation of exogenous formate. Claassens et al. [19] has successfully introduced a fully functional synthetic rGlyP into *Cupriavidus necator* which replaces the native ATP‐inefficient Calvin cycle for formatotrophic growth. Gonzalez et al. [20] has engineered *Saccharomyces cerevisiae* for growth on formate, achieving thereby glycine biosynthesis from formate and CO_2_ via the rGlyP. However, as shown for *C. pasteurianum*, potential imbalance or burdens in cell growth and energy and redox metabolism caused by the integration of an artificial GCS need to be better solved. To develop an engineered strain that can grow rapidly on formate and CO_2_ without glucose supplementation, further improvement of formate and CO_2_ assimilation ability into pyruvate is needed.

At present, the mostly used approach to improve the flux of the rGlyP pathway is the over‐expression of native enzymes and the introduction of heterologous genes which come from other species. Bang and Lee [13] have shown that the rGlyP efficiency is improved mainly by enhancing the catalytic activity of GCS, which is the central part of this pathway. They also pointed out that improvement of the affinities of the corresponding enzymes toward CO_2_ and NH_3_ can be a promising solution to enhance the rGlyP flux.

**PRACTICAL APPLICATION**

The greenhouse gas carbon dioxide (CO_2_) is the largest contributor to global warming and climate change. With the aim of reducing CO_2_ and realizing a carbon‐neutral economy, reconstruction of natural and synthetic CO_2_ fixation pathways in heterotrophic microorganisms is a promising way to solve this problem. Currently, the reductive glycine pathway (rGlyP) as one of the CO_2_ fixation pathways has been successfully introduced into microbial hosts of industrial interest, but its flux still does not meet the requirements of rapid growth of microorganisms. Enhancing the efficiency of glycine synthesis catalyzed by the reverse reaction of the glycine cleavage system (GCS) is of great significance for improving the rGlyP flux. Till now, there have been few studies on this aspect either in vitro or in vivo. To this end, several factors possibly affecting the catalytic efficiency of GCS on the synthesis of glycine were investigated in vitro in the present study.

GCS is a multi‐component protein system catalyzing a reversible reaction in terms of glycine cleavage or synthesis. In the direction of glycine synthesis, it functions only in a small group of anaerobic microbes such as *Clostridium acidiurici* [21], *Eubacterium acidaminophilum* [22] and *Arthrobacter globiform* [23, 24, 25], which can utilize CO_2_, ammonium, 5,10‐CH_2_‐THF and NADH to synthesis glycine. The four GCS protein components are not associated tightly to form a complex, it is more like a “system” than a “complex” [26]. The overall glycine synthesis reaction proceeds in three steps (Figure 1) in which lipoylated H protein (H_lip_, also called H_ox_ due to the disulfide bond on the dithiolane ring of the attached lipoyl group) is first reduced by NADH to generate dihydrolipoyl H protein (H_red_) by interaction with the L protein; T protein then catalyzes the formation of aminomethyl‐bearing H protein (H_int_) using 5,10‐CH_2_‐THF and ammonia as the donor of the methylene group and amino group, respectively; Afterwards, glycine is synthesized by P protein from CO_2_ and the aminomethyl group carried by H_int_, and the regenerated H_ox_ returns to the next cycle of glycine synthesis. Although GCS is so important in the one‐carbon assimilation pathway, there are relatively few quantitative studies on the GCS. The majority of GCS studies carried out between 1960s and 1980s were mainly in a qualitative manner. Most of the studies aimed at how to isolate and purify GCS proteins from plant cells [27, 28, 29], animal livers [30] and microorganisms [31, 32], and the examination of the reaction mechanisms. Recently, our group has studied the catalytic mechanism related to interactions between H protein and other GCS proteins. Zhang et al. [33] presented a detailed molecular dynamics (MD) analysis of the interactions within the *E. coli* H‐T protein complex that governs the induced release of the aminomethyl lipoate arm from a protected state in the cavity of H protein.

In the present study, we overexpressed and purified the individual GCS component proteins from *E. coli* and successfully reconstructed a multi‐enzyme reaction system that catalyzes the synthesis of glycine from formaldehyde and NH_4_HCO_3_ (or NaHCO_3_ and NH_4_Cl instead) in vitro. Because 5,10‐CH_2_‐THF is susceptible to oxidative breakdown and easy to decompose into formaldehyde and THF under acidic and neutral conditions, it is difficult to handle the reaction process by directly use 5,10‐CH_2_‐THF as substrate. To circumvent this problem, 5,10‐CH_2_‐THF was in situ synthesized through the spontaneous condensation of formaldehyde and THF in the reaction mixture. NH_4_HCO_3_ (or NaHCO_3_ and NH_4_Cl instead) was used to provide both CO_2_ and NH_3_ for the glycine synthesis reaction. Quantitative information about glycine synthesis in vitro was obtained by systematically examining factors that may affect the rate of glycine synthesis, including substrate concentration, ratio of the four GCS protein components, and the presence of reductant and metal ions. It is anticipated that this in vitro study should provide useful hints for improving the activity of GCS in vivo.

## 2 MATERIALS AND METHODS

### 2.1 Materials and reagents

All chemicals used in this study were of analytical grade or higher quality. Pyridoxal 5‐phosphate monohydrate (PLP), dithiothreitol (DTT), NADH, and NAD^+^ were purchased from Yuanye Bio‐Technology (Shanghai, China). β‐Mercaptoethanol (β‐ME) and lipoic acid were obtained from Aladdin (Shanghai, China). Tetrahydrofolate (THF) was obtained from Sigma‐Aldrich (St. Louis, MO, USA). Phusion high‐fidelity DNA polymerase and FastDigest restriction enzymes were purchased from Thermo‐Fisher Scientific (Pittsburgh, PA, USA). T4 DNA ligase was purchased from New England Biolabs (Ipswich, MA, USA). DNA extraction kit and gel extraction kit were purchased from Promega (Madison, WI, USA). The pET‐22b(+) and pET28a(+) vectors were purchased from Novagen (Darmstadt, Germany). All synthetic oligonucleotides were ordered from GeneWiz (Suzhou, China). Competent cells of *E. coli* TOP10 and *E. coli* BL21 (DE3) were purchased from WEIDI Ltd. (Shanghai, China). Ni‐NTA resin was purchased from Genscript (Nanjing, China). BCA protein assay kit was purchased from SolarBio (Beijing, China).

### 2.2 Bacterial strains and plasmids construction

Strains and plasmids used in this study are listed in Table 1. *E. coli* Top10 strain was used for the construction of plasmids. *E. coli* BL21 (DE3) cells were used as the host of enzyme overexpression and purification. The genes coding for P protein, T protein, H protein and L protein from *E. coli* were amplified by PCR from the genomic *E. coli* MG1655 cells. The DNA fragments were double‐digested with NdeI and XhoI, and separately cloned into the plasmid pET‐28a (+) to yield pET28a‐P, pET28a‐T, pET28a‐H, and pET28a‐L, respectively.

**TABLE 1:** Strains and plasmids used in this study for the overexpression of GCS proteins

	Description	Reference
*E. coli* strains		
Top 10	Host for plasmid cloning	WEIDI Ltd.
BL21 (DE3)	Host for protein overexpression	WEIDI Ltd.
Plasmids		
pET28a (+)	Vector for protein overexpression	Novagen
pET28a‐P	pET28a vector containing P protein gene (NCBI No. WP_112929453.1)	This study
pET28a‐T	pET28a vector containing T protein gene (NCBI No. WP_099356926.1)	This study
pET28a‐H	pET28a vector containing H protein gene (NCBI No. WP_001295377.1)	This study
pET28a‐L	pET28a vector containing L protein gene (NCBI No. WP_110826218.1)	This study

### 2.3 Protein expression and purification

The resulting constructs (pET28a‐P, pET28a‐T, pET28a‐H and pET28a‐L) were transformed into *E. coli* BL21 (DE3) cells. Cells carrying the plasmid pET28a‐H were grown in LB medium containing 50 μg/mL kanamycin and 200 μM lipoate acid at 37°C [34, 35]. Other *E. coli* cells containing the plasmids pET28a‐P, pET28a‐T and pET28a‐L, respectively, were grown in LB medium supplemented with 50 μg/mL kanamycin at 37°C. When the optical density at 600 nm reached 0.8, gene expression was induced by adding 0.2 mM isopropyl β‐d‐thiogalactopyranoside (IPTG). The culture was continued for a further 12 h at 30°C. Cells were harvested by centrifugation at 4700 rpm for 20 min, washed twice with Tris‐HCl buffer (50 mM, pH 7.5), re‐suspended in a lysis buffer (pH 7.5) containing 10 mM imidazole, 0.3 M NaCl and 50 mM Tris‐HCl and then disrupted by ultrasonication. The supernatant was loaded onto a Ni^2+^‐NTA column pre‐equilibrated with the lysis buffer. After protein binding, the column was washed with a wash buffer containing 30 mM imidazole, 0.3 M NaCl and 50 mM Tris‐HCl, and then eluted with an elution buffer containing 300 mM imidazole, 0.3 M NaCl and 50 mM Tris‐HCl. Fractions containing the targeting protein were pooled, and imidazole was removed by dialysis with 50 mM Tris‐HCl (pH 7.5) containing 10% glycerol. The purified protein was checked by sodium dodecyl sulfate polyacrylamide gel electrophoresis (SDS‐PAGE, 12%) and the protein concentration was determined by using BCA protein assay kit. Finally, aliquots of each purified protein were stored at −80°C.

### 2.4 Glycine synthase activity assay

The reverse GCS reaction for glycine synthesis was either carried out at 37°C under rigid anaerobic conditions in an anaerobic chamber (COY Laboratory Products) or under aerobic conditions by supplementing the reaction mixture with 20 mM of the reductant DTT or β‐ME. The reaction mixture contained 50 mM Tris‐HCl (pH 7.5), 0.5 mM THF, 2 mM formaldehyde, 25 μM PLP, 5 mM NADH, 50 mM NH_4_HCO_3_ (or 50 mM NaHCO_3_ and 50 mM NH_4_Cl instead), 5 μM P protein, 5 μM T protein, 5 μM L protein and 10 μM H protein. Glycine concentration in the reaction mixture was determined by HPLC after pre‐column dansyl chloride derivatization. To this end, 40 μL of a reaction mixture was mixed with 160 μL of 0.2 M NaHCO_3_ and 200 μL of 20 mM dansyl chloride in acetonitrile. Derivatization was carried out at 30°C for 30 min. The samples were analyzed on a Shimadzu LC‐2030C system equipped with a Shim‐pack GIST C18 column (5 μm, 4.6 × 250 mm) at 35℃ and a flow rate of 0.8 mL/min with a mobile phase composed of 25% acetonitrile and 75% of 20 mM potassium phosphate buffer (pH 6.0). The eluate was monitored at 254 nm using a photo‐diode array detector (PDA).

### 2.5 Glycine cleavage activity assay

The overall glycine cleavage activity was determined spectrophotometrically at 37°C by measuring glycine‐dependent NADH formation using a multimode plate reader (PerkinElmer, USA). The reaction mixture for the glycine cleavage reaction contained Tris‐HCl (50 mM, pH 7.5), 0.5 mM THF, 20 mM DTT, 25 μM PLP, 5 mM NAD^+^, 5 μM P protein, 5 μM T protein, 5 μM L protein and 10 μM H protein. After premixing and centrifugation, the reaction was initiated by the addition of 50 mM glycine.

### 2.6 Examining the cause of precipitation

Precipitation was observed in the mixture for the glycine synthesis reaction containing 0.5 mM THF, 20 mM DTT, 20 mM formaldehyde, 25 μM PLP, 5 mM NADH, and 50 mM NH_4_HCO_3_ but without any GCS proteins. To find out the causes of the precipitation experiments were carried out based on the principle of reducing one substrate at a time, as shown in Table 2.

**TABLE 2:** Experimental set‐ups (Groups 1–5) to examine the cause of precipitate formation in the reaction mixture during the glycine synthesis reaction

Component	G1	G2	G3	G4	G5
NH_4_HCO_3_	+	+	+	+	–
NADH	+	+	+	–	+
THF	+	+	–	+	+
PLP	+	–	+	+	+
Formaldehyde	–	+	+	+	+

### 2.7 Effects of metal ions on glycine synthase activity

The effects of metal ions (Mg^2+^, Mn^2+^, Fe^2+^, Co^2+^, Ni^2+^, Cu^2+^, Zn^2+^, Ba^2+^, Na^+^, Cs^+^) on the glycine synthesis activity of GCS were assayed by determining the glycine formation using HPLC. The components of the reaction mixture were the same as specified in the Section 2.4, except for the addition of individual metal ions. The glycine synthesis activity of GCS determined without the presence of any of the metal ions mentioned above was set at 100% as reference for comparison.

## 3 RESULTS AND DISCUSSION

### 3.1 Effects of the ratio of GCS component proteins on glycine synthesis rate

**FIGURE 2:**
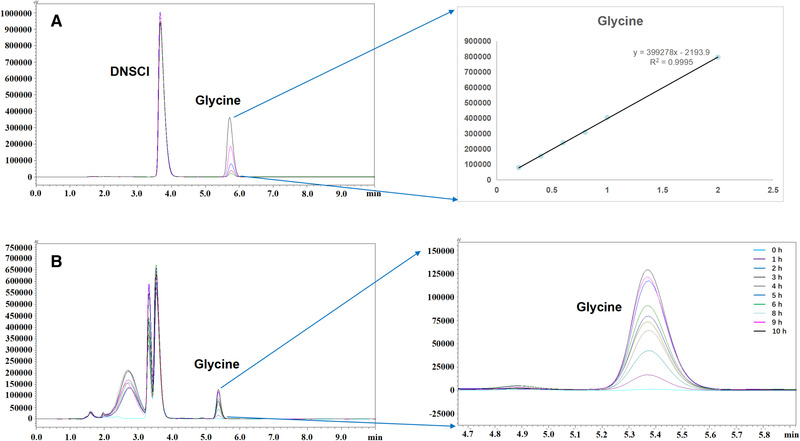
HPLC quantification of glycine after derivatization with dansylchloride (DNSCl). (A) Chromatograms of glycine standards and the external calibration curve. (B) Chromatograms of GCS reaction mixtures showing glycine formation after given reaction times

In order to gain more insight into the glycine synthesis catalyzed by GCS, we set out to construct a cell‐free multi‐enzyme catalytic system to learn how to improve the rate of the glycine synthesis reaction, a possible limiting factor of the rGlyP. To this end, we first expressed the four GCS proteins individually in *E. coli* BL21 (DE3) and purified them on Ni^2+^‐NTA column. A proof‐of‐concept experiment was then performed for the synthesis of glycine from formaldehyde and NH_4_HCO_3_. By HPLC monitoring the gradual accumulation of glycine during the proceeding of the reaction, the functionality of the constructed GCS reaction system was successfully confirmed (Figure 2).

After the proof‐of‐concept experiment, the effects of the ratio of the GCS component proteins and the concentration of different substrates on the glycine synthesis reaction rate were studied. First, for the construction of a GCS in vitro reaction system we needed to find the proper ratios of the four GCS proteins. In this regard, no relevant studies in *E. coli* are available. We only found a work from Oliver et al. [36] who studied the ratio of the GCS component proteins in pea leaf mitochondria by quantitative ELISA assays. Their results show that an approximate ratio of the four GCS proteins is 2 P protein dimers: 27 H protein monomers: 9 T protein monomers: 1 L protein dimer (corresponding to a P:H:T:L ration of 4:27:9:2), indicating a high ratio of H protein to the other three GCS component proteins. Therefore, we chose to start with a ratio of P:H:T:L = 1:2:1:1 to study the glycine synthesis, and then varied the ratio to see the influences of the different GCS proteins.

**FIGURE 3:**
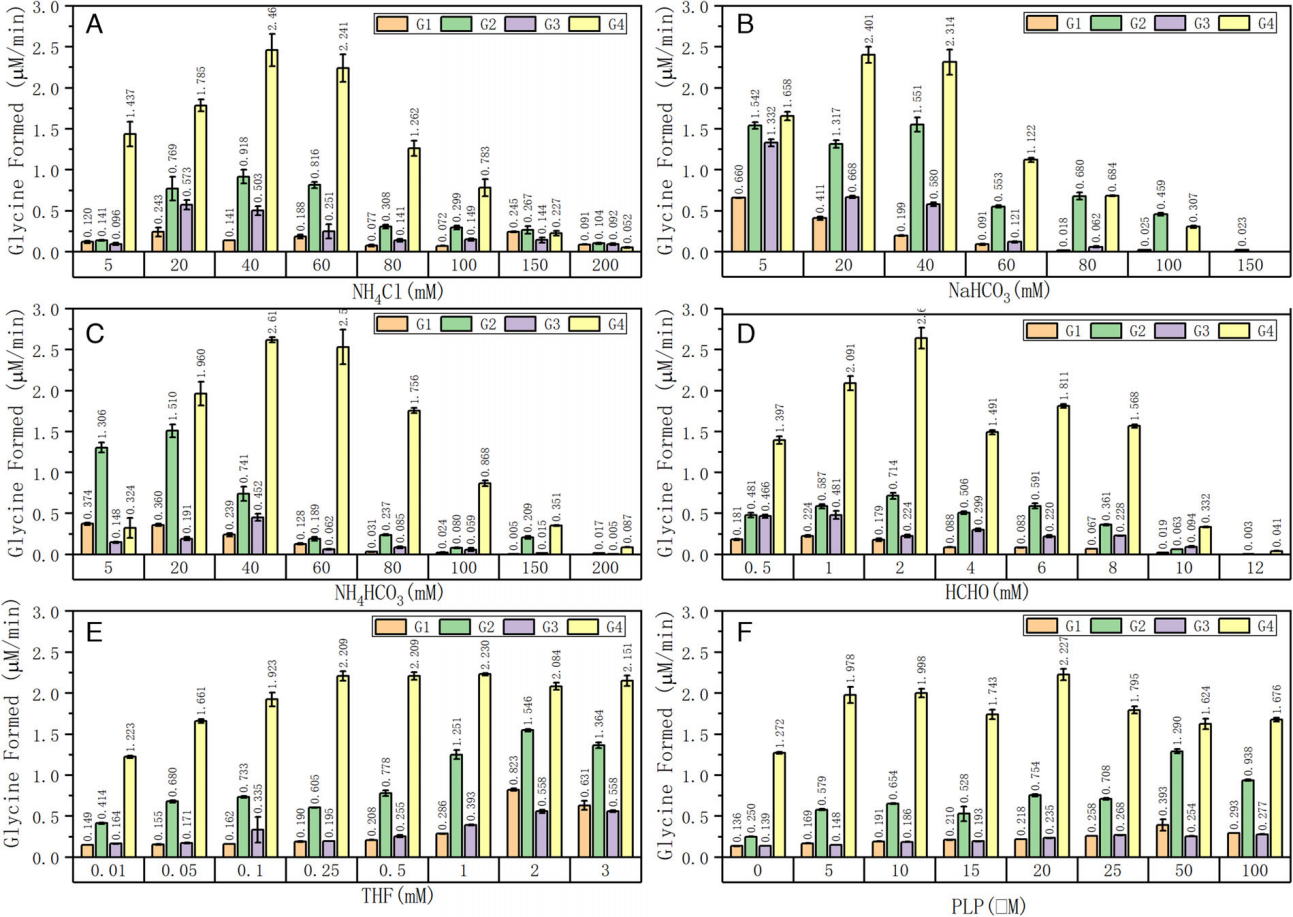
Effects of the ratios of GCS component proteins and the concentrations of different substrates on the overall glycine synthesis reaction rate under anaerobic conditions (A) NH_4_Cl, (B) NaHCO_3_, (C) NH_4_HCO_3_, (D) formaldehyde, (E) THF and (F) PLP. The substrates CO_2_ and NH_3_ for the in vitro glycine synthesis by reverse GCS are supplemented either in the combined form of NH_4_HCO_3_ or in the separated forms of NH_4_Cl + NaHCO_3_. The concentrations (μM) of GCS component proteins used in the assay groups G1 to G4 are given in Table 3, corresponding to a ratio of the GCS component proteins (P:H:T:L) of 1:2:1:1 (G1), 4:2:1:1 (G2), 1:2:0.1:1 (G3) and 1:8:1:1 (G4)

As shown in Figure 3, we found that the ratio of the GCS component proteins has a great influence on the initial reaction rate of glycine synthesis. The concentrations of the GCS component proteins used in the groups G1 to G4 are shown in Table 3. A general trend can be observed that the rate of glycine synthesis is G4 > G2 > G3 ≈ G1. When taking G1 as the control group, increasing the concentration of P protein from 5 μM in the G1 group to 20 μM in the G2 group leads to a significant increase of the reaction rate. This clearly indicates that the carboxylation reaction catalyzed by P protein is a key step determining the glycine synthesis rate. Comparing the influence of T protein, reducing the concentration of T protein from 5 μM in the G1 group to 0.5 μM in the G3 group is advantageous and enhances the reaction rate in the majority cases (Figure 3A,B,D). In the G4 group, the concentration of H protein is 40 μM compared to 10 μM in the G1 group. This increase brings about the most striking enhancement in the glycine synthesis rate. It unambiguously points out that H protein has a huge influence on the rate of glycine synthesis under the given in vitro reaction conditions. Unlike P, T and H proteins, variation in the concentration of L protein has no significant effect on glycine synthesis (data not shown).

**TABLE 3:** Concentrations (μM) of GCS component proteins used in the assay groups G1 to G4 to investigate the influence of the ratio of GCS component proteins on the rate of glycine synthesis

Assay group	P protein	T protein	L protein	H protein
G1	5	5	5	10
G2	20	5	5	10
G3	5	0.5	5	10
G4	5	5	5	**40**

### 3.2 Effects of the concentration of different substrates on glycine synthesis rate

We used either NH_4_Cl and NaHCO_3_ together or NH_4_HCO_3_ alone to provide the two substrates NH_3_ and CO_2_ for the glycine synthesis. Comparing the results shown in Figure 3A‐D, it is to see that high concentrations of not only formaldehyde but also NH_4_Cl, NaHCO_3_ and NH_4_HCO_3_ are detrimental to glycine synthesis, indicating substrate inhibition. We speculate that high ionic strength is not conducive to the carboxylation reaction, resulting in the reduction of reaction rate [37, 38]. The most obvious differences occur in the low substrate concentration range especially at the concentration of 5 mM. Taking G1 as control, in the case of using NH_4_HCO_3_ (5 mM NH_4_Cl + 5 mM NaHCO_3_, molar ratio of NH_3_/CO_2_ is 1:1) as substrate (Figure 3C), increasing P protein from 5 to 20 μM is favorable, most likely due to enhanced carboxylation reaction. However, increasing the concentration of NaHCO_3_ to change the NH_3_/CO_2_ ratio to 1:50 (5 mM NH_4_Cl + 50 mM NaHCO_3_) wipes out this positive effect and leads to overall decreased rates of glycine synthesis (Figure 3A). On the contrary, increasing the concentration of NH_4_Cl to change the NH_3_/CO_2_ ratio to 50:1 (5 mM NaHCO_3_ + 50 mM NH_4_Cl) enhances glycine synthesis (Figure 3B). This means, under the given reaction conditions a higher ratio of NH_3_ to CO_2_ is, in general, advantageous for enhancing the rate of glycine synthesis. Especially perplexing is that although NH_3_ is the direct substrate of the aminomethyl transfer reaction catalyzed by T protein in glycine synthesis, reducing T protein concentration from 5 to 0.5 μM (G3) to lower the ratio of T protein in the GCS complex is even advantageous for glycine synthesis under the situation of higher NH_3_/CO_2_ ratio. Thus, the above results reveal a quite complex interdependence between the ratio of GCS component proteins and the ratio of the two key substrates and therefore the dynamic complexity of this multi‐enzyme multi‐substrate GCS system, even under the in vitro conditions. Comparing the case at NH_3_/CO_2_ ratio of 1:1, it is quite interesting that increasing H protein concentration from 10 to 40 μM brings about strongly enhanced rate of glycine synthesis both for the increased and decreased NH_3_/CO_2_ ratios. The unknown reason may lie in the fact that H protein as the shuttle protein is special in the GCS, as it is not only a component of the GCS complex but also an intermediate (in its three forms) in the GCS cascade reaction.

Another substrate 5,10‐CH_2_‐THF was in situ generated through an non‐enzymatic spontaneous condensation reaction of formaldehyde with THF [39]. As shown in Figure 3D, increasing the concentration of formaldehyde from 0.5 to 2 mM increases the initial glycine synthesis rate. However, it is noted that formaldehyde as a toxic compound can cross‐link with enzymes to reduce their catalytic activity, leading to decrease in the initial reaction rate when the concentration of formaldehyde exceeds 2 mM, and a complete inhibition of glycine synthesis over a concentration of 12 mM. Figure 3E depicts that the initial glycine synthesis rate increases to a maximum of about 2.2 μM/min with the increase of THF concentration but levels off after the THF concentration reaches about 0.25 mM. It was previously reported that a high concentration of THF could inhibit the reaction rate in THF‐dependent enzymatic reaction [23] which was, however, not obvious till a THF concentration of 3 mM in our experiments. Figure 3F shows the effect of PLP on the initial glycine synthesis rate. It is interesting to see that this cofactor of P protein seems to be not necessary, and the reaction can occur without adding PLP in the reaction mixture. A possible reason behind this irrational result is that a part of P protein is already bound with PLP during the process of its overexpression in *E. coli* [40]. Nevertheless, adding additional 5 mM PLP increases the reaction rate from about 1.3 to 2.0 μM/min in the G2 group, which contains 20 μM (four times of other groups) P protein, but further increase in PLP concentration seems to be not necessary and does not result in further increase of the reaction rate.

### 3.3 Determination of the rate‐limiting component in glycine synthase

**FIGURE 4:**
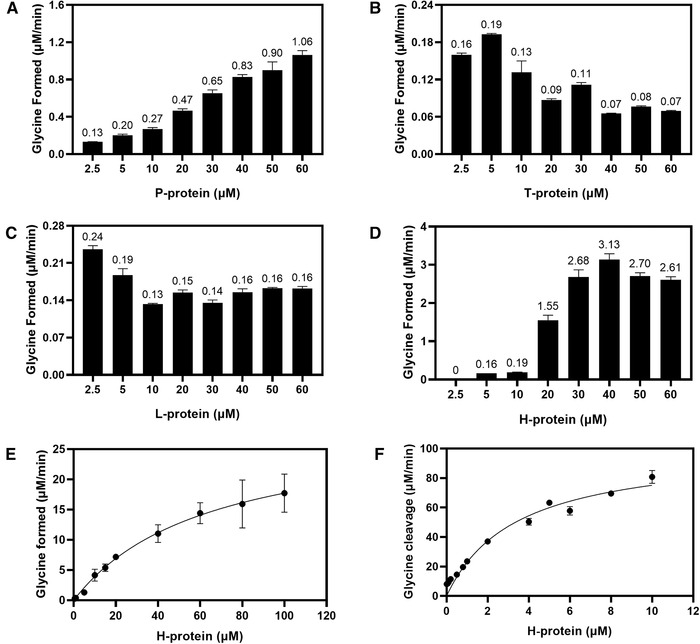
Effects of concentrations of different GCS proteins (A) P protein, (B) H protein, (C) T protein, (D) L protein on the overall glycine synthesis reaction rate under anaerobic conditions. Other reaction components are as given in the Section 2.4. Glycine synthesis (E) and glycine cleavage (F) catalyzed by GCS under aerobic conditions in response to the concentration change of H protein. Other reaction components are as given in Sections 2.4 and 2.5

Based on the above experimental results, we decided to systematically study the effect of the concentration ratio of the four GCS component proteins on the glycine synthesis rate. As shown in Figure 4A, by keeping the concentrations of H, T, and L proteins at 10, 5, and 5 μM, respectively, gradually increasing the concentration of P protein up to 60 μM (corresponding to a ratio of P:H:T:L = 12:2:1:1) leads to a continuous increase in the glycine synthesis rate, indicating that the carboxylation reaction is a rate limiting step of the overall glycine synthesis reaction. This is in contrast to the molar ratio of the GCS proteins being P:H:T:L = 4:27:9:2 in pea leaf mitochondria determined by Oliver et al. [36]. In plants, glycine created from an unwanted byproduct of the Calvin cycle is cleaved by GCS, and P protein functions in the direction of decarboxylation. Therefore, it can be speculated that the carboxylation reaction for glycine synthesis needs more P protein than the decarboxylation reaction for glycine cleavage. This reminds us of a recent interesting discovery that the reverse of citric acid cycle is mainly dependent on a high level of the enzyme citrate synthase which allows autotrophic CO_2_ fixation in a primordial atmosphere [41, 42].

The rate of glycine synthesis increases by about 26% with the increase of the concentration of T protein from 0.5 to 5 μM Figure 4B. However, with the further increase of the concentration of T protein, the rate of glycine synthesis decreases, indicating that excessive T protein inhibits the reaction rate in the direction of glycine synthesis. Figure 4C shows that increasing the concentration of L protein does not improve the rate of glycine synthesis, indicating that the reduction of lipoamide group of H‐protein catalyzed by L‐protein is not the rate‐limiting step in determining the rate of glycine synthesis.

In agreement with the results shown in Figure 3 and compared with the other three GCS proteins, increase in the concentration of H protein is most effective in enhancing the glycine synthesis rate (Figure 4D). The largest rise (about eight folds) occurs when the concentration of H protein increases from 10 to 20 μM, corresponding to a molar ratio of P:H:T:L = 1:4:1:1. With further increasing the concentration of H protein to 40 μM the rate of glycine synthesis continues to increase to a maximum of 3.13 μM/min. As shuttle protein, H protein plays the role of bridging P protein, T protein and L protein in the GCS. We were then curious to know how the concentration of H protein would affect glycine cleavage in comparison to glycine synthesis. As shown in Figure 4E,F, under the conditions of keeping the concentrations of the other three proteins constant, both the rates of glycine synthesis and glycine cleavage increase significantly with increasing the concentration of H protein. However, the results of nonlinear fitting show that the *V_max_* in the direction of cleavage is 3.5 times of that in the direction of synthesis. This means, a much higher ratio of H protein to the other GCS component proteins will be required to achieve a comparable reaction rate for glycine synthesis than for glycine cleavage. Therefore, a possible strategy to increase the flux of the rGlyP in *E. coli* can be a stronger overexpression of H protein.

In addition, we noted that the maximum rate of glycine synthesis shown in Figure 4D is less than that in Figure 4E. This difference is attributed to the fact that the experiment in Figure 4D was carried out under anaerobic condition, while the experiment in Figure 4E was performed under aerobic condition using reductant DTT to prevent THF from oxidation. Therefore, whether DTT may act as an enhancer to promote the glycine synthesis reaction deserves a close look.

### 3.4 Effect of reductants on the glycine synthesis rate

**FIGURE 5:**
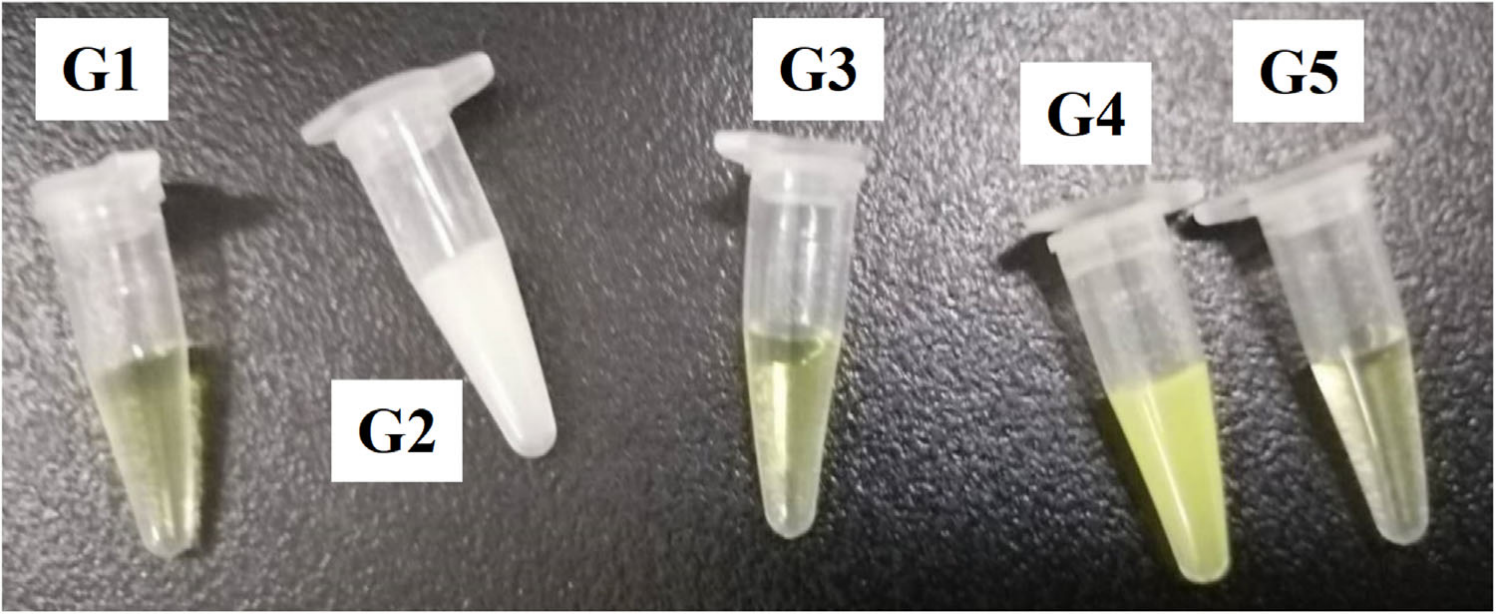
Precipitate formation in the reaction mixture used for the glycine synthesis reaction. The composition of the reaction mixtures of G1 to G5 is summarized in Table 2

We used DTT to protect THF and 5,10‐CH_2_‐THF (formed from the spontaneous condensation of THF with formaldehyde as a reaction substrate) and therefore enabled the glycine synthesis reaction to be carried out under aerobic conditions. When formaldehyde concentration used was higher than 20 mM, we observed precipitation in the reaction mixture 5 min after mixing all the reaction components. After centrifugation to remove the precipitate, the glycine synthesis reaction continues in the supernatant as evidenced by the increased accumulation of glycine in the supernatant over time. There are two possible reasons for precipitation: one is the partial denaturation of GCS proteins caused by high concentration of formaldehyde; the other one is an unknown chemical reaction between the reaction components. We found that the precipitate in the reaction mixture was not a typical white flocculent precipitate what we normally observed by protein aggregation due to denaturation. Therefore, we speculated that the precipitation may be caused by chemical reaction between the reaction components. We carried out a group of experiments, with each group lacking one of the reaction components we reckoned that they might contribute to the precipitation (Table 2). Precipitation was not found in G1, G3 and G5, but in G2 and G4 (Figure 5). What G2 and G4 have in common that differentiates them from G1, G3 and G5 is that both have the three components formaldehyde, THF and NH_4_HCO_3_, which may be responsible for the precipitation. In the following experiment for confirmation, when formaldehyde, THF and NH_4_HCO_3_ were mixed together, precipitation was indeed observed. A close examination further revealed that the precipitation is caused by the interaction of formaldehyde, DTT and NH_4_^+^.

**FIGURE 6:**
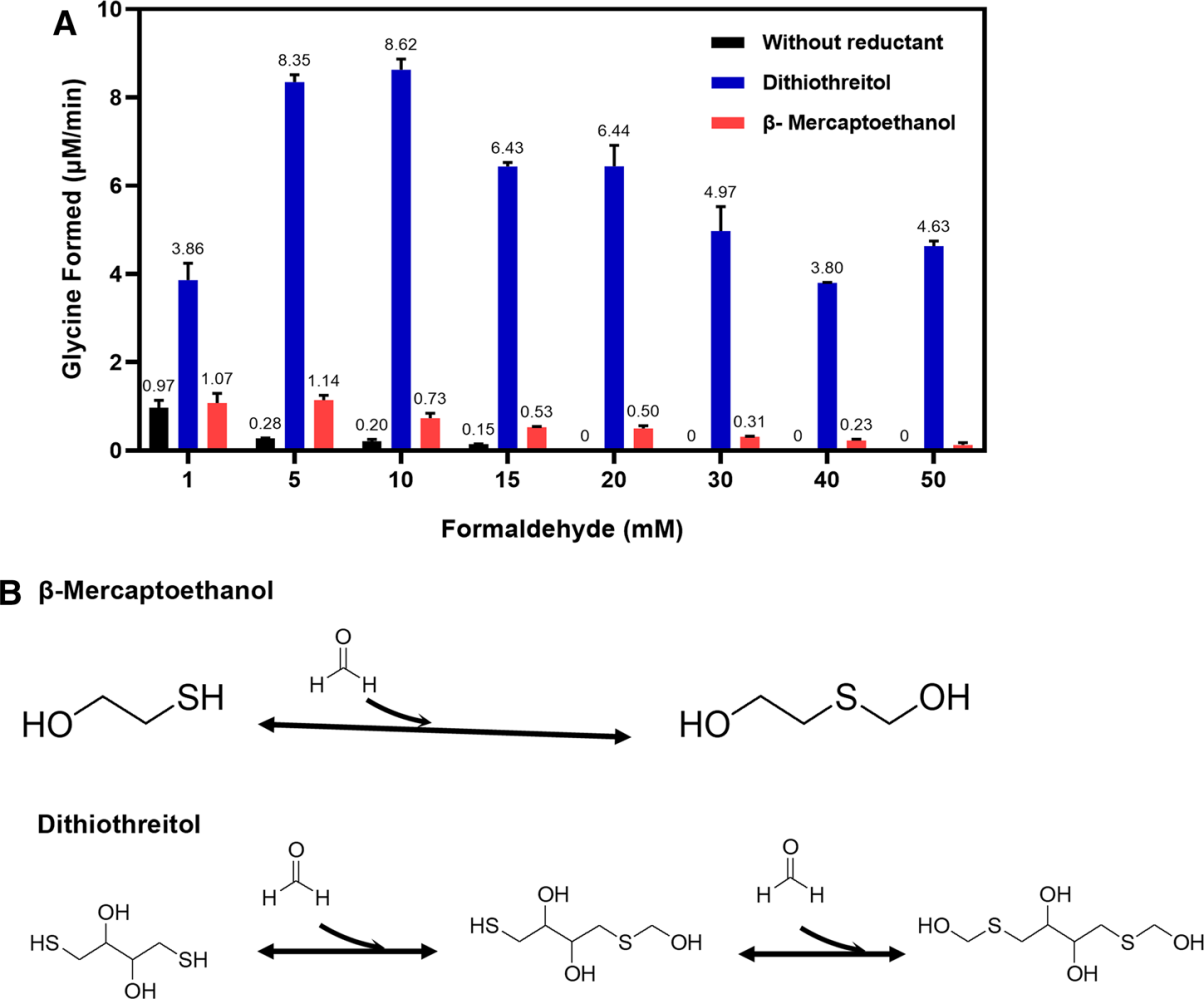
(A) Effect of formaldehyde concentration on the rate of glycine synthesis in the absence or presence of different reductants under anaerobic conditions. “Without reductant” refers to the glycine synthesis reaction mixture specified in Section 2.4 without any reductant added; “Dithiothreitol” and “β‐Mercaptoethanol” refer to glycine synthesis reaction mixtures containing dithiothreitol and β‐mercaptoethanol, respectively, while other reaction components are the same as in “Without reductant.” (B) Hypothesized mechanisms of β‐mercaptoethanol and dithiothreitol improving the tolerance of GCS enzymes to formaldehyde

As the addition of DTT will cause precipitation in the reaction mixture, we tried to use β‐mercaptoethanol (β‐ME), which does not cause precipitation, to avoid oxidative degradation of THF under aerobic condition. Figure 6A shows the effects of different concentrations of formaldehyde on glycine synthesis rate with or without reductant under anaerobic conditions. The glycine synthesis rate decreases with the increase of formaldehyde concentration in the group “without reductant”. When the concentration of formaldehyde exceeds 15 mM, glycine formation could not be detected in the reaction mixture, demonstrating thereby the toxicity of formaldehyde towards GCS proteins. In comparison, the addition of a reductant DTT or β‐ME in the reaction mixture improves the tolerance of GCS proteins to formaldehyde. The reason why the addition of a reductant can improve the tolerance of GCS to formaldehyde is shown in Figure 6B. The reductant containing sulfhydryl group undergoes a reversible condensation reaction with formaldehyde to reduce the toxicity of formaldehyde. Since this experiment was carried out under anaerobic conditions, which should actually avoid the oxidative degradation of THF and therefore does not require a reductant, the enhanced glycine synthesis rate can be attributed to the protection of GCS proteins by DTT or β‐ME against the toxicity of formaldehyde rather than to the protection of THF by the reductants.

However, DTT demonstrates much better protective effect than β‐ME. In fact, β‐ME is not the right choice for replacing DTT in this case. We speculate that the presence of DTT not only prevents the toxicity of formaldehyde, but improves the activity of P protein. According to the findings of Hasse et al. [40], the activity of P protein from the *Cyanobacterium synechocystis* sp. PCC 6803 is regulated by redox homeostasis, and the crystal structure of the P protein shows the C terminus being locked in a closed conformation by a disulfide bond between Cys^972^ and Cys^353^. The presence of this disulfide bridge isolates the active site from solvent and hinders the binding of PLP and glycine in the active site. The reduction of the disulfide bond releases the C terminus and allows the access of PLP and glycine to the active site. Thus, the addition of DTT can actively reduce the disulfide bond and therefore activate P protein. Of course, it must be pointed out that the increase in the glycine synthesis rate may also be due to the fact that the disulfide bond‐reductants DTT or TCEP [43, 44] allow the reduction of the disulfide bond on the dithiolane ring of the lipoyl group attached on H_ox_ and therefore taking up the function of the L protein without even the requirement for NADH in the in vitro glycine synthesis reaction system.

### 3.5 Effect of metal ions on glycine synthesis

As presented in Table 4, a series of experiments was performed to test the effect of various metal ions in two concentrations of 1 and 5 mM, respectively, on the GCS activity of glycine synthesis. The glycine synthesis reaction catalyzed by GCS was significantly inhibited by various monovalent and divalent cations such as Co^2+^, Ni^2+^, Zn^2+^, Ba^2+^, and Cs^+^. Among them, Co^2+^, Ni^2+^, and Zn^2+^ completely suppress the activity of GCS, while Ba^2+^ and Cs^+^ inhibited the activity of the GCS to a certain extent. On the contrary, the additions of Mg^2+^, Mn^2+^, Fe^2+^, Cu^2+^, and Na^+^ at the low concentration of 1 mM enhance the glycine synthesis rate. However, increase of the concentration of these metal ions to 5 mM already led to the decrease of the glycine synthesis rate to different degrees. Therefore, we cannot rule out that higher Na^+^ concentration may also contribute to a certain extent to the general downward trend of the glycine synthesis rate shown in Figure 3B with increased concentration of NaHCO_3_. However, at least up to a concentration of 40 mM of NaHCO_3_ there is no stark influence either from Na^+^ or from HCO_3_^–^. The obviously decreased glycine synthesis rate above the concentration of 60 mM of NaHCO_3_ is more likely due to the limited carboxylation activity of P protein. The mechanisms behind the effects of the metal ions on the synthesis of glycine catalyzed by the GCS remain unclear. The study of the overall glycine synthesis reaction doesn't allow us to accurately understand the specific binding sites of metal ions. Kikuchi et al. [45] suggested that some divalent ions with inhibitory effect on the glycine synthesis reaction may be due to the bonding of metal ions with the remaining free thiol group in the aminomethyl‐bearing H protein (H_int_) and hindering thereby the carboxylation reaction catalyzed by P protein to form glycine. Individual metal ions may have different affinities for H_int_ and this difference may be responsible for the observed different degrees of inhibition by individual metal ions. Further research needs to study the glycine synthesis reaction catalyzed by the GCS step by step, e.g. studying the effect of metal ions on the reaction steps catalyzed by P protein, T protein and L protein, separately.

**TABLE 4:** Influences of metal ions on the activity of GCS

Metal ions	Concentration (mM)	Relative activity (%)
None	0	100.0 ± 13.5^a^
	0	100.0 ± 6.5^b^
MgCl_2_	1	117.6 ± 17.7
	5	32.9 ± 7.9
MnCl_2_	1	147.8 ± 28.1
	5	124.4 ± 5.1
FeSO_4_	1	164.0 ± 22.3
	5	97.9 ± 12.0
CoCl_2_	1	0.0
	5	0.0
NiCl_2_	1	0.0
	5	0.0
CuCl_2_	1	161.7 ± 28.0
	5	0.0
ZnCl_2_	1	0.0
	5	0.0
BaCl_2_	1	88.3 ± 10.1
	5	47.2 ± 10.1
NaCl	1	159.1 ± 18.2
	5	120.8 ± 7.7
CsCl	1	55.0 ± 5.3
	5	80.4 ± 2.7

^a^ Result of the control group used for the assay groups of 1 mM metal ion.

^b^ Result of the control group used for the assay groups of 5 mM metal ion.

## 4 CONCLUDING REMARKS

In this paper, we successfully constructed an in vitro GCS‐catalyzed glycine synthesis system for kinetic study of the GCS‐catalyzed glycine synthesis. We studied some key factors that may affect the rate of the glycine synthesis. To this end, the effects of substrate concentrations on the glycine synthesis rate under different ratios of the four GCS component proteins were examined. The experimental results showed that P protein and especially H protein have significant effects on the overall glycine synthesis reaction, as higher concentration ratios of these two proteins to other GCS proteins are favorable for enhancing the rate of glycine synthesis. On the contrary, increasing the concentration of L protein has no effect on the rate of glycine synthesis, and a high concentration of T protein even negatively affects the synthesis reaction, resulting in decreased reaction rate. In addition, the disulfide reducing agent DTT was found to positively influence the rate of glycine synthesis, probably not only due to its role in preventing THF and 5,10‐CH_2_‐THF from oxidation but also its functions in activating P protein and taking up the role of L protein in the non‐enzymatic reduction of H_ox_ to H_red_. Some metal ions (Mg^2+^, Mn^2+^, Fe^2+^, Cu^2+^ and Na^+^) are shown to improve the rate of glycine synthesis due to yet unknown reasons. These findings may provide some guidance for improving the efficiency of GCS‐catalyzed glycine synthesis as an important part of the rGlyP for use of C1 carbons in vivo.

As discussed previously, the plant GCS has a relatively low ratio of the P protein versus other subunits for the functionality of glycine cleavage by GCS, while our in vitro study of glycine synthesis by GCS indicates that a higher ratio of P protein is probably needed for the reverse GCS reaction. It is not clear if this also holds true for in vivo microbial glycine synthesis. At present, the complete rGlyP has been successfully introduced into *E. coli* and *C. necator* by the method of genetic engineering. These engineered microorganisms contain natural GCS operons. Unfortunately, so far, the ratios of the component proteins of these native GCS in these microorganisms have not been examined so far. It is therefore of great importance to gain such information by means of e.g. proteomics so as to guide the adjustment of the expression ratios of the GCS component proteins in industrial hosts with the goal of increasing the flux of the rGlyP.

In our in vitro study, we found that GCS has low catalytic activity for glycine synthesis (*K_cat_* value ∼0.1 s^–1^). Researches on glycine synthesis catalyzed by GCS were mainly carried out in the 1960s‐1980s in which the overall reaction in the glycine synthesis direction was studied by C^14^ radio labeling. We calculated the enzyme activity in these publications and found that the rate of glycine synthesis reaction is indeed very slow. In the work of Kochi and Kikuchi published in 1976 [25], the *K_cat_* in the direction of glycine synthesis is calculated to be barely 0.036 s^–1^, which is even lower that what we obtained in this study, namely ∼0.1 s^–1^. It is therefore tempting to speculate that glycine synthesis reaction catalyzed by GCS can be the rate‐limiting step of the rGlyP. Bang and Lee [13] have reported that they have improved the rGlyP flux mainly by enhancing the glycine synthesis reaction catalyzed by GCS. Due to the discrepancy between the in vivo and in vitro environments (e.g. because of mostly unknown in vivo concentrations of substrates/intermediates/cofactors and concentration/ratio/activity of enzymes), the GCS activity measured in vitro may not reflect the real situation in vivo. Nevertheless, in vitro studies are relatively fast and convenient which can help us screen out conditions that increase the rate of glycine synthesis reaction. For example, we use it for in vitro determination of the glycine synthase activity affected by different H protein mutants.

In this study, we only determined the overall GCS activity in glycine synthesis (by HPLC quantification of glycine formation) and glycine cleavage (by spectrometric minoring NADH formation) to intuitively understand the influence of various factors on the overall GCS reaction, especially in the direction of glycine synthesis. However, it is necessary to develop suitable methods for the characterization of the single reaction steps catalyzed by the individual GCS enzymes so that it would be also possible to study how various factors affect each of the GCS reaction steps [46]. So far, the individual GCS component proteins are mainly studied by determining their activities through isotope labeling (P protein activity assay) or by spectrophotometry (L protein activity assay) [47], as well as their interactions through crystallographic analysis [46]. Development of novel analytical methods for the study of the kinetics and reaction mechanisms of individual GCS proteins and their interactions may help deepen our understanding of GCS for the purpose of engineering GCS to obtain highly efficient rGlyP. Our experimental studies revealed that among the four GCS proteins H protein has the greatest influence on the glycine synthesis reaction. Therefore, alternative to using H protein at high concentration, mutation of H protein to enhance its function can be also an effective way to further increase the glycine synthesis rate.

## CONFLICT OF INTEREST

The authors have declared no conflict of interest.

## Data Availability

Major data generated and analyzed during this study are included in the article. Other datasets generated and analyzed during the study are available from the corresponding author on reasonable request.
